# A Systematic Review of Diagnostic Biomarkers of COPD Exacerbation

**DOI:** 10.1371/journal.pone.0158843

**Published:** 2016-07-19

**Authors:** Yu-Wei Roy Chen, Janice M. Leung, Don D. Sin

**Affiliations:** Centre for Heart Lung Innovation, Institute for Heart Lung Health at St. Paul’s Hospital & Department of Medicine, Division of Respiratory Medicine, University of British Columbia, Vancouver, BC, Canada; Helmholtz Zentrum München, GERMANY

## Abstract

The aims of this systematic review were to determine which blood-based molecules have been evaluated as possible biomarkers to diagnose chronic obstructive pulmonary disease (COPD) exacerbations (AECOPD) and to ascertain the quality of these biomarker publications. Patients of interest were those that have been diagnosed with COPD. MEDLINE, EMBASE, and CINAHL databases were searched systematically through February 2015 for publications relating to AECOPD diagnostic biomarkers. We used a modified guideline for the REporting of tumor MARKer Studies (mREMARK) to assess study quality. Additional components of quality included the reporting of findings in a replication cohort and the use of receiver-operating characteristics area-under-the curve statistics in evaluating performance. 59 studies were included, in which the most studied biomarkers were C-reactive protein (CRP), interleukin-6 (IL-6), and tumor necrosis factor-alpha (TNF-α). CRP showed consistent elevations in AECOPD compared to control subjects, while IL-6 and TNF-α had variable statistical significance and results. mREMARK scores ranged from 6 to 18 (median score of 13). 12 articles reported ROC analyses and only one study employed a replication cohort to confirm biomarker performance. Studies of AECOPD diagnostic biomarkers remain inconsistent in their reporting, with few studies employing ROC analyses and even fewer demonstrating replication in independent cohorts.

## Introduction

Chronic obstructive pulmonary disease (COPD) is a debilitating disease that is characterized by reduced lung function, breathlessness, decreased productivity, and poor quality of life [1]. Currently, COPD is the only major cause of mortality with a rising death rate and it is estimated that by 2030 COPD will become the fourth leading cause of death worldwide [2,3]. The natural history of COPD is often marked by periodic exacerbations in which symptoms of breathlessness and sputum production worsen acutely, resulting in emergency room visits and hospitalizations [1,4,5]. In Canada, acute exacerbations of COPD (AECOPD) account for the highest rate of hospital admissions and repeat hospitalizations [6], with an estimated economic burden amounting to $4.5 billion dollars each year in direct and indirect costs [7].

Owing to their heterogeneity and the lack of available diagnostic laboratory tests, AECOPD are often diagnosed based on clinical gestalt, which is subjective and variable within and across physicians. Forced expiratory volume in the first second of expiration (FEV1) has conventionally been used to guide therapy in stable COPD; however, it is a poor indicator of a patient’s exacerbation status [1]. Instead, biomarkers are biological molecules that may better reflect disease activity and fluctuate in accordance with disease state, while representing biologically plausible pathways [8]. Theoretically, as readily available point-of-care tests that can supplement clinical data, they could provide a more objective determination of a patient’s health status before, during, and after an AECOPD event [9–11]. While levels of these biomarkers may be altered when comparing stable COPD patients to normal controls [12], further disturbances may be observed in the acute setting of an exacerbation. Biomarkers could further allow physicians to provide personalized care for each patient by tailoring targeted therapies based on biomarker levels, thus avoiding unnecessary side effects of prolonged exposure to drugs or conversely incompletely treating an AECOPD. For instance, certain biomarkers could potentially point to a bacterial or viral origin, thus guiding appropriate therapy [13].

There have been numerous articles published over the past decade, which have focused on the discovery and assessment of biomarkers in relation to AECOPD [14]. Similarly, there have been a wide variety of sample types that have been collected for this purpose including exhaled breath condensate, sputum, nasal wash, blood, bronchoalveolar lavage, and lung biopsies. In this review, we have focused our attention on blood-based biomarkers to diagnose exacerbations. This type of sample has clear advantages that make clinical translation facile including non-invasiveness, ease of collection, widespread availability of laboratories that can procure and process these samples, and the ability to standardize measurements for most assays. The aims of this systematic review are to determine which plasma or serum molecules have been evaluated (and published) as possible biomarkers to diagnose AECOPD and to ascertain the quality of these publications with the view of determining which molecules, if any, have the greatest potential for clinical translation.

## Methods

### Study population

Our population of interest was defined as COPD patients of any age and gender, who had experienced exacerbations, and as a consequence, required medical attention and admission to a hospital for treatment. We included studies that examined patients longitudinally (i.e. onset of AECOPD versus convalescence), and also those that were cross-sectional (i.e. AECOPD versus stable COPD). Studies that focused on exacerbation biomarkers to guide therapeutic treatments were excluded. The biomarkers, which we subsequently reviewed after study selection, were categorized and described in terms of their use in the diagnosis of AECOPD onset.

### Literature search and article selection

We employed our search strategy in accordance with the Preferred Reporting Items for Systemic Reviews and Meta-Analysis (PRISMA) guidelines (S1 PRISMA Checklist), as well as PRISMA for abstracts [15,16]. We searched articles in MEDLINE (1966–2015), EMBASE (1980–2015), CINAHL (1982–2015), and Cochrane databases by using specific Medical Subject Headings (MeSH) terms. We used Elsevier ScienceDirect as an additional source. The MeSH terms included a combination of: chronic obstructive pulmonary disease or COPD, exacerbations, acute exacerbation, or AECOPD, biomarkers or biological markers, diagnosis or diagnostic, and blood or serum or plasma (a detailed list of MeSH terms is provided in S1 Table). Two authors (YWRC and JML) independently screened the titles and abstracts based on the articles’ relevance to our MeSH terms, with disagreements resolved by iteration and consensus. Primary articles that were published in English, focused on human subjects (animal studies were excluded), and performed analysis on blood specimens were retained. Entries of review articles, conference abstracts, book chapters, editorials, or original articles that performed biomarker assessments on sputum samples, or breath condensate were excluded. References from selected articles were also reviewed to ensure the inclusion of all relevant articles.

### Data extraction and study quality assessment

To assess the quality of publications reporting biomarkers, we first screened the 59 relevant original papers for the two important analytic components of biomarker studies, as recommended by Sin and colleagues [17]. These components included: 1) the use of receiver operating characteristics (ROC) area-under-the curve (AUC) statistics or equivalent in reporting the performance of the biomarker as a diagnostic tool for AECOPD, and 2) the reporting of biomarker findings in a replication cohort or sub-cohort of the parent study. We additionally used the Guidelines for the REporting of Tumor MARKer Studies (REMARK) to rank these selected publications (see S2 Table for a detailed REMARK checklist) [18]. Because REMARK was created for oncology studies, we modified the criteria to enable use for AECOPD (resulting in a modified or mREMARK score). For instance, where the guidelines mentioned recommendations pertaining to tumor biomarkers (in points 13 and 15 of S2 Table), we simply replaced these terms with "AECOPD biomarkers". In cases where the guidelines referred to standard prognostic variables (in points 14 and 17 of S2 Table), we replaced this concept with lung function measurements, which have significant prognostic value in COPD. A collection of mREMARK scores were then collated and ranked. Higher mREMARK scores were considered reflective of a higher quality study, with the maximum achievable mREMARK score being 20.

## Results

### Search results

**Fig 1:**
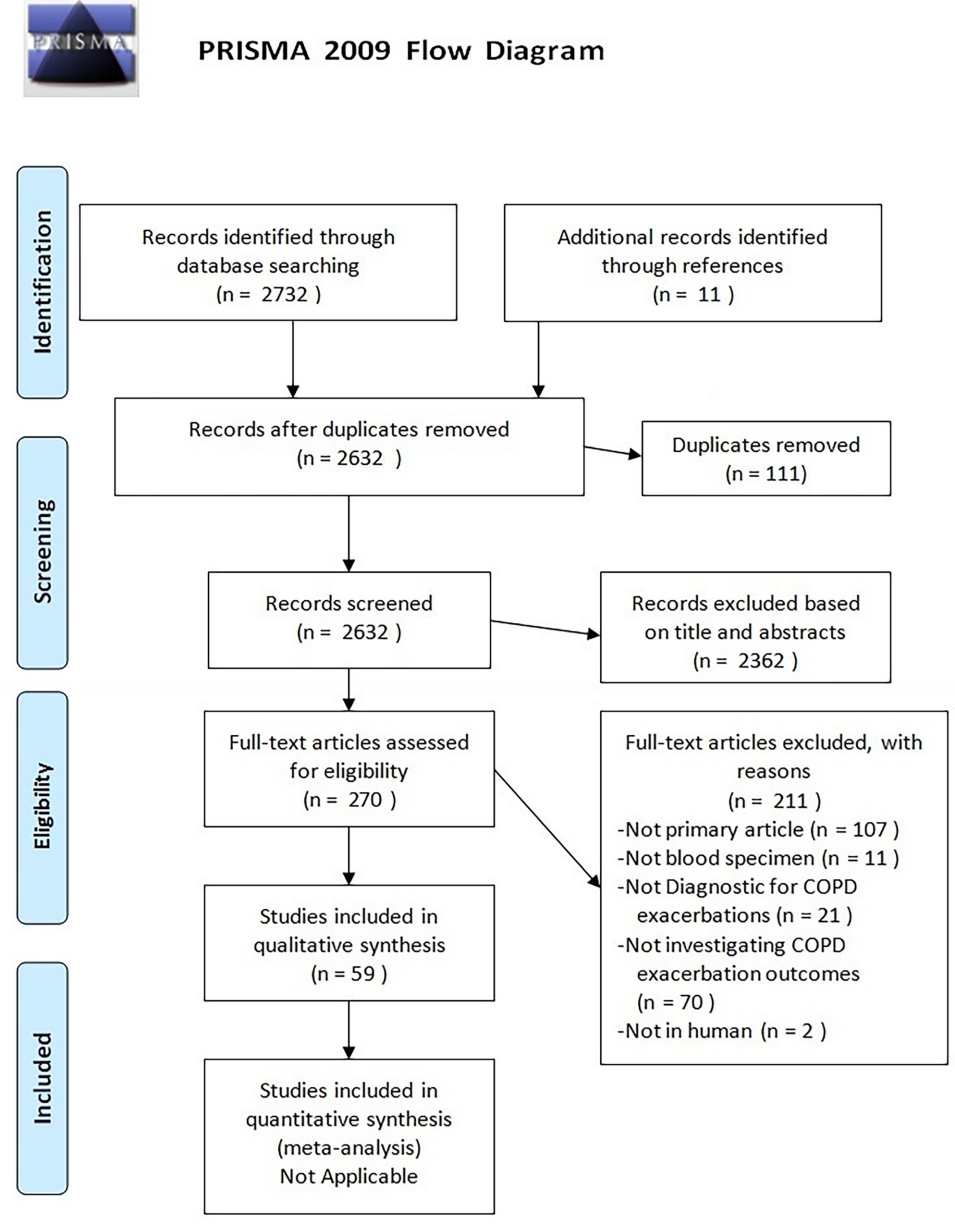
PRISMA flow diagram of study screening and selection. PRISMA flow diagram used in study selection and screening. 59 studies were included for review whereas the rest of the studies were excluded.

The initial search revealed a total of 2,732 studies, of which 111 were duplicate articles (see Fig 1). We also excluded 2,362 other articles by screening titles and abstracts of these articles because they were not relevant to this analysis, leaving 270 articles eligible for full-text review. Among these articles, 211 articles were excluded for reasons that are outlined in Fig 1. In total, 59 studies were included in the qualitative analysis. A flow diagram of study screening and selection is shown in Fig 1.

### Study characteristics

The study characteristics are displayed in Table 1 and the patient characteristics are listed in S3 Table. The definitions used by all of the studies pertaining to COPD diagnosis, AECOPD, and stable COPD are listed in S4 Table. The majority of the studies (53 out of 59) defined COPD diagnosis based on the Global initiative for chronic Obstructive Lung Disease (GOLD) criteria (FEV1/FVC <70%), and a bronchodilator effect of <12% in either the FEV1 or FVC [1]. 33 out of 59 studies defined an AECOPD based on worsening of symptoms including dyspnea, cough, or sputum production, which led to the intensification in the use of maintenance medications and/or institution of “rescue” medications [1,5]. Definitions of stable COPD were highly variable among the 59 studies, with duration free of exacerbation ranging from 3 weeks to 3 months (see S4 Table). The majority of the studies (12 out of 59) defined stable COPD as being free of exacerbation in the preceding 4 weeks.

**Table 1:** Study characteristics of 59 publications included in the review arranged by the latest published year.

**Reference**	**Year**	**Country**	**Single or Multi-centre**	**Biomarkers Tested**	**Cross-Sectional Assessment**	**Longitudinal Assessment**
Andelid, K., et al. [19]	2015	Sweden	S	CRP, MPO, Neutrophil Elastase, WBC	Yes	Yes
Gumus, A., et al. [20]	2015	Turkey	S	CRP, fibrinogen, SuPAR	Yes	Yes
Chang, C., Yao, W. [21]	2014	China	S	CRP, IL-6	No	Yes
Chang, C., et al. [22]	2014	China	S	CRP, IL-6, WBC	No	Yes
Fattouh, M. Alkady, O. [23]	2014	Egypt	S	CRP, Fibrinogen, WBC	Yes	Yes
Johansson, S.L., et al. [24]	2014	Denmark	M	CRP, MFAP4, SP-D, WBC	Yes	Yes
Labib, S., et al. [25]	2014	Egypt	S	Desmosine	Yes	Yes
Lee, S.J., et al. [26]	2014	Korea	S	Osteopontin	Yes	Yes
Liu, H.C., et al. [27]	2014	Taiwan	S	IL-8, IL-17	Yes	No
Liu, Y., et al. [28]	2014	China	S	CD34+ cells, CRP, MMP-9, NT-proBNP	Yes	No
Meng, D.Q., et al. [29]	2014	China	S	Adrenomedullin, CRP, WBC	Yes	Yes
Nikolakopoulou, S., et al. [30]	2014	Greece	S	Angiopoietin-2, CRP	No	Yes
Nishimura, K., et al. [31]	2014	Japan	S	BNP	Yes	Yes
Omar, M.M., et al. [32]	2014	Egypt	S	Adiponectin	Yes	Yes
Oraby, S.S., et al. [33]	2014	Egypt	S	Adiponectin	Yes	No
Urban, M.H., et al. [34]	2014	Austria	S	sRAGE	No	Yes
Zhang, Y., et al. [35]	2014	China	S	CRP, Fibrinogen, HMGB1, sRAGE	No	Yes
Zhao, Y.F., et al. [36]	2014	China	S	Copeptin, CRP, Procalcitonin	No	Yes
Adnan, A.M., et al. [37]	2013	Syria	S	ECP, Eotaxin/CCL11, IL-8	Yes	No
Carter, R.I., et al. [38]	2013	UK	S	Aα-Val^360^	No	Yes
Gao, P., et al. [39]	2013	China	S	CRP, IL-6, MMP-9, Serum Amyloid-A	Yes	No
Jin, Q., et al. [40]	2013	China	S	RBP4	Yes	No
Mohamed, N.A., et al. [41]	2013	Egypt	S	Adiponectin, CRP, IL-6, IL-8, TNF-α	Yes	No
Patel, A.R.C., et al. [42]	2013	UK	M	CRP, fibrinogen, NT-proBNP, Troponin T	No	Yes
Scherr, A., et al. [43]	2013	Switzerland	S	Pancreatic stone protein/regenerating protein	Yes	No
Shoukry, A., et al. [44]	2013	Egypt	S	IL-6, TNF-α, Thyroid hormone T3, T4, TSH	Yes	No
Stanojkovic, I., et al. [45]	2013	Serbia	S	Beta-crosslaps, CRP, MMP-9, TIMP-1	Yes	Yes
Chen, H., et al. [46]	2012	China	S	507 inflammatory mediators	Yes	No
Falsey, A.R., et al. [47]	2012	USA	S	Procalcitonin	Yes	Yes
Huang, J., et al. [48]	2012	UK	M	Desmosine	Yes	No
Ju, C.R., et al. [49]	2012	China	S	CRP, SP-D	Yes	Yes
Koczulla, A.R., et al. [50]	2012	Germany	S	Alpha-1 antitrypsin, CRP, Procalcitonin, WBC	Yes	No
Kwiatkowska, S., et al. [51]	2012	Poland	S	MMP-9, TIMP-1	Yes	Yes
Marcun, R., et al. [52]	2012	Slovenia	S	NT-proBNP, Troponin T	No	Yes
Mohamed, K.H., et al. [53]	2012	Egypt	S	CRP, ESR, Procalcitonin, WBC	Yes	No
Pazarli, A.C., et al. [54]	2012	Turkey	S	CRP, ESR, Procalcitonin, WBC	Yes	No
Rohde, G., et al. [55]	2012	Germany	S	sTREM-1	Yes	Yes
Shaker, A., et al. [56]	2012	Egypt	S	FSH, IGF-1, LH, Testosterone	Yes	Yes
Yerkovich, S.T., et al. [57]	2012	Australia	S	Anti-VP1 IgG_1_, IL-21	Yes	No
Bafadhel, M., et al. [13]	2011	UK	S	24 biomarkers (including CCL4, CCL17, CRP, CXCL11, ECP, Eosinophil % count, IFNγ, IL-5, IL-6, IP-10, Neopterin, Procalcitonin, Serum Amyloid-A, SP-D, TNFR1, TNFR2)	No	Yes
Chen, H., et al. [58]	2011	China	S	40 inflammatory mediators (including betacellulin, CCL17, CCL22, CCL23/MPIF-1, CCL25, CCL27, CCL28, CXCL11, IL-9, MCP-3, MCP-4, osteopontin)	Yes	Yes
Lacoma, A., et al. [59]	2011	Spain	M	MR-proANP	Yes	Yes
Lacoma, A., et al. [60]	2011	Spain	M	CRP, Neopterin, Procalcitonin	Yes	Yes
Lim, S.C., et al. [61]	2011	Korea	S	IL-6, IL-8, TNF-α, T-Lymphocyte Apoptosis	Yes	No
Markoulaki, D., et al. [62]	2011	Greece	M	CRP, EPO, Fibrinogen, Hgb, IL-6, TNF-α	No	Yes
Krommidas, G., et al. [63]	2010	Greece	M	Adiponectin, CRP, IL-6, Leptin, TNF-α	No	Yes
Quint, J.K., et al. [64]	2010	UK	M	CRP, IL-6, IP-10	Yes	Yes
Koutsokera, A., et al. [65]	2009	Greece	S	CRP, Fibrinogen, IL-6, Serum Amyloid-A, TNF-α	No	Yes
Kythreotis, P., et al. [66]	2009	Greece	S	IGF-1, IL-1β, IL-6, IL-8, Leptin, TNF-α	Yes	Yes
Shakoori, T.A., et al. [67]	2009	Parkistan	S	SP-D	Yes	No
Karadag, F., et al. [68]	2008	Turkey	S	IL-6, NO, TNF-α	Yes	Yes
Stolz, D., et al. [69]	2008	Switzerland	S	CRP, BNP, Procalcitonin	No	Yes
Groenewegen, K.H., et al. [70]	2007	Netherlands	S	BPI, IL-6, sIL-1RII, sTNFR55, sTNFR75, TEAC	Yes	Yes
Perera, W. R., et al. [71]	2007	UK	S	CRP, IL-6	No	Yes
Pinto-Plata, V.M., et al. [72]	2007	USA	S	IL-6, IL-8, LTB4, SLPI, TNF-α	No	Yes
Hurst, J.R., et al. [73]	2006	UK	Multi Centre	36 biomarkers (including Adiponectin, CRP, CCL4, CCL5, CCL23/MPIF-1, Eotaxin-2, IFNγ, IL-1Ra, IL-6, IL-8, IL-17, IP-10, MCP-1, MMP-9, MPO, PARC/CCL18, sICAM-1, TGF-α, TIMP-1, TNFR1, TNFR2)	No	Yes
Phua, J., et al. [74]	2006	Spain	S	sTREM-1	Yes	No
Roland, M., et al. [75]	2001	UK	S	Endothelin-1	No	Yes
Fiorini, G., et al. [76]	2000	Italy	S	ECP, IgE, MPO	Yes	No

Note: For studies that included more than 10 biomarkers, not all markers are listed. Abbreviations: S = S, M = multi centre. **Biomarker abbreviations: Aα-Val**^**360**^ **= fibrinogen cleavage product, Anti-VP1 IgG**_**1**_ **= immunoglobulin G1 antibody against viral protein 1, BNP = brain natriuretic peptide, BPI = bactericidal permeability increasing protein, CD = cluster of differentiation, CCL = chemokine C-C motif ligand, CXCL = chemokine C-X-C motif ligand, ECP = eosinophil cationic protein, EPO = erythropoietin, ESR = erythrocyte sedimentation rate, FE = frequent exacerbators, FSH = follicle stimulating hormone, GPx = erythrocytic glutathione peroxidase, Hgb, = hemoglobin, HMGB = high mobility group box, IFN = interferon, IG = immunoglobulin, IGF = insulin-like growth factor, IL = interleukin, IP = interferon-γ inducible protein, LH = luteinizing hormone, LTB4 = leukotriene B4, MCP = monocyte chemoattractant protein, MFAP = microfibrillar associated protein, MMP = matrix metallopeptidase, MPIF = myeloid progenitor inhibitory factor, MPO = myeloperoxidase, MR-proANP = Mid-regional prohormone of atrial natriuretic peptide, NE = non-exacerbators, NO = nitric oxide, NT-proBNP = amino-terminal of the prohormone of brain natriuretic peptide, PARC = pulmonary and activation-regulated chemokine, RBP = retinol-binding proteins, sICAM = soluble intercellular adhesion molecule, sIL-1R = soluble interleukin 1 receptor, SLPI = secretory leukocyte protease inhibitor, SP = surfactant protein, sTNFR = soluble tumor necrosis factor receptors, sRAGE = soluble receptor for advanced glycation end-products, sTREM = soluble triggering receptor expressed on myeloid cells, suPAR = soluble urokinase-type plasminogen activator receptor, TEAC = Trolox equivalent antioxidant capacity, TGF = transforming growth factor, TIMP = tissue inhibitors of metalloproteinase, TNF = tumor necrosis factor, T3 = Triiodothyronine, T4 = thyroxine, TSH = thyroid stimulating hormone, and WBC = white blood cell.**

41 studies evaluated biomarkers longitudinally in the same patients at onset of and recovery from AECOPD. 42 studies evaluated biomarkers cross-sectionally between patients with AECOPD and stable COPD patients, and/or healthy controls. Approximately half of the studies included in the review were performed in Europe (47%), with the United Kingdom being the most prevalent location. Most of the studies were single-centre based (86%) and all were prospective in design. 81% of the studies had a relatively small study size (defined as less than 100 exacerbating patients). The total number of patients included in the 59 studies was 5,431, with a range of 9 to 333 COPD patients per study. Patients were mostly males, and the mean age of all COPD participants were 64 years with a mean FEV1% of approximately 47%.

### Biomarkers for the diagnosis of AECOPD

In total, 134 distinct biomarkers were measured, with one additional study measuring 507 inflammatory mediators using an antibody microarray [46]. Biomarkers evaluated covered a wide range of molecules: acute phase reactants such as C-reactive protein (CRP), erythrocyte sedimentation rate (ESR) and fibrinogen; cytokines such as interleukin (IL)-6, IL-8, and TNF-α; molecules of cardiac origin such as brain natriuretic peptide (BNP); molecules involved in collagen formation such as matrix metalloproteinase (MMP)-9; and molecules involved in fatty acid processing such as adiponectin. The most commonly studied biomarker was CRP, followed by IL-6 and TNF-α (see S5 Table). The use of CRP as a biomarker was investigated in 28 studies, in which 26 of these reported a statistically significant increase in concentration during AECOPD versus stable COPD and/or healthy controls. The CRP assays used were highly variable; five studies used an immunonephelometric assay, four studies used an immunoturbidimetric assay, and three studies used an immune latex agglutination assay. Despite these differences in techniques, the reported CRP results were congruent with each other. IL-6 was investigated in 18 studies, in which 13 showed significant increases during AECOPD versus stable COPD. Three studies also reported increased IL-6 levels during AECOPD, but the statistical analysis was either non-significant or not reported. TNF-α was investigated in ten studies, but with variable statistical significance. Seven out of ten studies reported significant increases in TNF-α concentrations during AECOPD compared to stable COPD, whereas two did not show statistical significance and one showed borderline statistical significance. Nevertheless, TNF-α concentrations were numerically higher in AECOPD compared with stable COPD or healthy controls. The measurement methods used for both IL-6 and TNF-α were all immuno-based assays, such as enzyme-linked immunosorbent assay (ELISA).

51 biomarkers that were investigated only in a single study are detailed in S6 Table along with statistical comparisons. Approximately half of these biomarkers were reported with an increased concentration during AECOPD, and the other half reported the opposite. 18 of these biomarkers were not statistically different between levels during AECOPD versus stable state.

### Study quality assessment

Out of the 59 articles, only 12 (20%) reported an ROC analysis (see Table 2). Eight of the 12 studies used an ROC analysis to characterize biomarker performance in the diagnosis of AECOPD. Two studies performed ROC analyses to characterize biomarker performance in predicting AECOPD mortality. One study used the ROC to determine which patients would require antibiotics and one study to discriminate between mild, moderate, and severe AECOPD. The articles had a median mREMARK score of 13/20 with a range from 6 to 18/20; the detailed breakdown of the scores is tabulated in S7 Table. Only one article (2%), by Bafadhel et al, utilized a second, independent cohort to replicate biomarker performance [13]. This study, which had the highest mREMARK score at 18, included 145 AECOPD patients, and used a combination of serum biomarkers and sputum biomarkers to classify patients into four distinct exacerbation phenotype clusters [13]. CRP, CXCL10 and peripheral eosinophil counts were found to be useful in distinguishing between bacterial-, virus-, and eosinophil-associated exacerbations, respectively. The AUC results were 0.65 (Confidence Interval (CI): 0.57–0.74) for CRP, 0.76 (CI: 0.67–0.86) for CXCL10, and 0.85 (CI: 0.78–0.93) for peripheral eosinophil count. Findings of bacterial-associated exacerbations via CRP were replicated with a comparable AUC of 0.70 (CI: 0.59–0.82). In addition, findings of virus-associated exacerbations via CXCL10 were also replicated with a comparable AUC of 0.65 (CI: 0.52–0.78).

**Table 2:** Selected publications from the review with Biomarker ROC analysis performance.

**Reference**	**Sample Size**	**Biomarker**	AUC (95% CI)*	**Replication**	**ROC Use**	**mREMARK Score (/20)**
Bafadhel M., et al. [13]	145 COPD	CRP	1) 0.65 (0.57–0.74) 4) 0.73 (0.61–0.85)	External	1) Discriminate bacterial AECOPD cluster 2) Discriminate viral AECOPD cluster 3) ROC for eosinophil associated exacerbations 4) Validation performance	18
		IL-6	1) 0.67 (0.58–0.76)			
		Serum Amyloid-A	1) 0.67 (0.58–0.76)			
		TNFR1	1) 0.62 (0.53–0.71)			
		TNFR2	1) 0.60 (0.50–0.70)			
		CXCL10	2) 0.76 (0.67–0.86) 4) 0.65 (0.52–0.78)			
		CXCL11	2) 0.67 (0.56–0.78)			
		IFNγ	2) 0.65 (0.54–0.75)			
		Peripheral Eosinophil %	3) 0.85 (0.78–0.93)			
		IL-5	3) 0.65 (0.55–0.76)			
		CCL17	3) 0.63 (0.53–0.73)			
Lacoma, A., et al. [60]	• 217 AECOPD • 46 Stable COPD	CRP	1) 0.53 (0.72–1.61) 2) 0.68 (0.65–1.76)	None	1) Discriminate bacterial AECOPD 2) Discriminate bacterial AECOPD with clinical symptoms	16
		Procalcitonin	1) 0.52 (0.71–1.38) 2) 0.66 (0.65–1.42)			
		Neopterin	1) 0.61 (0.39–0.90) 2) 0.70 (0.31–0.87)			
Stolz, D., et al. [69]	208 AECOPD	BNP	1) 0.55 (0.41–0.68) 2) 0.56 (0.45–0.66)	None	1) Discriminate 6-month mortality 2) Discriminate 2-year mortality	15
Jin, Q., et al. [40]	64 AECOPD	RBP4	0.88 (0.78–0.94)	None	Discriminate AECOPD patient mortality	15
Hurst, J.R., et al. [73]	90 AECOPD	CRP only	1) 0.66–0.80) 2) 0.88 (0.82–0.93)	None	1) Discriminate AECOPD versus Stable COPD 2) Discriminate AECOPD with CRP and one major symptom versus Stable COPD	14
		CRP, MMP-9, and MPIF-1	1) 0.75 (0.67–0.82)			
		All 36 biomarkers	1) 0.79 (0.73–0.86)			
Gumus, A., et al. [20]	43 AECOPD	suPAR	0.81 (0.72–0.90)	None	Discriminate AECOPD on day 1 versus day 7	13
		Fibrinogen	0.76 (0.66–0.86)			
		CRP	0.70 (0.58–0.81)			
Shakoori, T.A., et al. [67]	• 13 AECOPD• 14 Stable COPD• 54 Controls	SP-D	1) 0.76 (0.60–0.92) 2) 0.68 (0.48–0.89)	None	1) Discriminate AECOPD versus stable COPD and Controls 2) Discriminate AECOPD versus stable COPD	13
Falsey, A.R., et al. [47]	• 184 AECOPD• 56 Pneumonia• 16 Bacterial and viral AECOPD• 25 viral AECOPD	Procalcitonin	1) 0.76 (0.68–0.84) 2) 0.75 (0.67–0.82) 3) 0.70 (0.53–0.87)	None	1) Discriminate AECOPD versus pneumonia patients at day 1 2) Discriminate AECOPD versus pneumonia patients at day 2 3) Discriminate bacterial and viral AECOPD versus viral AECOPD alone	12
Quint, J.K., et al. [64]	72 AECOPD	IP-10 (CXCL10)	1) 0.78 (0.65–0.91) 2) 0.82 (0.74–0.90)	None	1) Discriminate HRV-positive AECOPD versus HRV-negative AECOPD 2) Discriminate HRV-positive AECOPD with coryzal symptoms versus HRV-negative AECOPD	11
Pazarli, A.C., et al. [54]	• 68 AECOPD• 50 Stable COPD	Procalcitonin	1) 0.89 (0.80–0.97) 2) 0.89 (0.82–0.97)	None	1) Discriminate mild versus moderate/severe exacerbation 2) Discriminate patients with NPPV versus without	10
Phua, J., et al. [74]	• 43 COPD• 72 Pneumonia• 35 Asthma• 63 Controls	sTREM-1	0.77 (0.70–0.84)	None	Discriminate patients requiring antibiotics versus those that do not	10
Adnan, A.M., et al. [37]	• 35 AECOPD• 30 Stable COPD• 23 Controls	Eotaxin	1) 0.70 2) 0.87	None	1) Discriminate AECOPD versus stable COPD 2) Discriminate Stable COPD versus Controls	6

* AUC provided for the specific ROC use.

Selected studies from the review that contained biomarker characteristics and ROC performances. Only studies that performed an ROC analysis were included in this table and those without a biomarker ROC analysis were not included. REMARK scores are assigned based on whether the studies met the 20 reporting recommendations. The table is arranged in descending order based on the REMARK scores. Abbreviations: AUC = area under the curve, CCL = chemokine C-C motif ligand, CI = confidence interval, CXCL = chemokine C-X-C motif ligand, ECP = eosinophil cationic protein, FE = frequent exacerbators, HRV = human rhinovirus, ICU = intensive care unit, IFN = interferon, IL = interleukin, IP = interferon-γ inducible protein, LVD = left ventricular dysfunction, MMP-9 = matrix metallopeptidase-9, MPIF-1 = myeloid progenitor inhibitory factor-1, N/A = not available, NPPV = non-invasive positive pressure ventilation, RBP = retinol-binding protein, REMARK = recommendations for tumor marker prognostic studies, ROC = receiver-operator characteristics, Sens = sensitivity, Spec = specificity, SP-D = surfactant protein-D, TNFR = tumor necrosis factor receptor.

## Discussion

Ideally, a clinically useful biomarker, whether for AECOPD or other diseases, should consistently and accurately reflect disease activity. The test should perform similarly in a variety of COPD patients and cohorts, while inflicting the least invasiveness on a patient. In this systematic review, we have identified 59 studies that have prospectively evaluated a wide range of blood-based biomarkers in the diagnosis of AECOPD; however, we found a number of deficiencies in the literature that have likely impeded the translation of these biomarkers into widespread clinical use. Very few of these studies reported performance of their biomarkers using an ROC analysis and even fewer replicated their findings in an external cohort. Along these lines, many biomarkers have only been tested in single centres, again raising the necessity for validation of these results. Moreover, the definitions employed for AECOPD and stable states were inconsistent across studies, making it difficult to assess overall biomarker performance. Until these gaps in the literature are addressed, a biomarker that can accurately and consistently diagnose AECOPD may be challenging to achieve. The best studied biomarkers to date have reflected inflammatory and cytokine pathways (CRP, IL-6, and TNF-α); however, of these, only CRP concentrations appeared to be consistently elevated in the AECOPD state compared to convalescence. Still, only four studies evaluating CRP used an ROC analysis and only one study employed a second validation cohort.

Given these discrepancies in the literature, minimum standard criteria implemented for every biomarker study may help to strengthen the quality of biomarker research. Currently, there is no standardized method of assessing the quality of COPD biomarker performance [17]. We propose here the use of the modified REMARK (mREMARK) score, derived from the oncology literature to better serve COPD. In the field of oncology, REMARK guidelines for biomarker studies have been in place since 2005 to facilitate the translation of biomarkers from discovery to clinical trials [18]. These detailed and rigorous criteria help to provide a realistic and reproducible performance assessment and importantly include the requirement that studies report estimated effects of biomarkers, validation, comparisons to standard prognostic variables, and transparent statistical methods. Although the REMARK checklist was originally developed to assess the quality of biomarker studies in oncology, we believe that with minimal modifications (“tumor” being replaced by “AECOPD” and standard tumor prognostic tools being replaced by lung function measurements), it may serve as an assessment tool in COPD exacerbation biomarker discovery. In accordance with mREMARK guidelines, very few among the selected studies in this systematic review would be deemed of “good” quality.

In addition to the mREMARK criteria, we recommend the use of ROC analyses and AUC statistics to objectively evaluate biomarker performance [77]. Such analyses confer distinct advantages over individual measures of sensitivity and specificity, and certainly over simple t-test statistics calculating significance between case and control biomarker levels (which can be falsely reassuring with a large enough sample size). For one, ROC analyses allow for an unbiased assessment of the best cut-off point for biomarker levels [78,79]. Second, the AUC statistic considers both sensitivity and specificity in reporting a test’s discriminative power, without regard to the prevalence of disease in specific populations [78,80]. It further allows an objective comparison of biomarker performance across studies and platforms whereby a biomarker with an AUC >0.85 is considered to have high accuracy while a biomarker with an AUC between 0.7 and 0.85 has borderline potential for clinical translation and warrants further refinement and validation. [17,81].

Furthermore, even in the 12 studies that reported an ROC analysis (some with remarkable AUC values), caution must be exercised when interpreting the results. The vast majority of these studies performed statistical analyses on discovery cohorts only without reproducing the performance in an external cohort. These studies run the risk of over-fitting their statistical model to the initial discovery cohort, particularly if the discovery cohort is small in size, and often times these initially optimistic findings may perform poorly in a new cohort. Data sharing across large cohorts has been proposed as one mechanism to counteract this obstacle [82], but in the absence of available external cohorts, methods such as cross-validation may provide an additional degree of validation and thus confidence that the results could be replicated in wider use. In this approach, the statistical model is applied to successive data sets in which one or more samples have been removed, and tested on the left out sample(s); the cross-validation AUC is therefore an estimate of the model’s discriminative power in the samples used. Types of cross-validation include leave-one-out cross-validation, *k-*fold cross-validation, and repeated random-split cross-validation. In leave-pair-out cross validation, a case and its corresponding control are left out in each iteration of the cross-validation [83,84]. In *k-*fold cross-validation, data are split into *k* subsets of equal size, with each one serving as the test set and the remaining *k*-1 subsets serving as the training set in successive iterations. In repeated random-split cross-validation, data are split into test and training sets randomly and repeatedly [84]. Given the risk of overly optimistic results with standard AUC statistics, we suggest that all future biomarker studies employ these cross-validation techniques, even in studies with available external validation cohorts.

There were a few limitations with our systematic review. First, the decision to use the mREMARK checklist to rank the selected studies in our review was arbitrary, as alluded to previously, but one made based on the fact that there are no alternative ranking methods for biomarker studies in COPD exacerbations. Nevertheless, the ranking scores via the mREMARK guidelines were objective measures and provided some guidance as to how to judge the quality of biomarker studies. Second, given the heterogeneity of biomarkers studied, we were not able to perform a meta-analysis on the results. However, we summarized the top three most studied biomarkers and their respective statistical significance to provide an overview. Last, we recognize there could be some potential publication bias, due to the fact that negative studies tend to be less published than positive studies.

In summary, while we found a number of studies that have evaluated potential candidate biomarkers for AECOPD diagnosis, we also identified a number of deficiencies in the COPD biomarker literature that make it difficult to fully evaluate the performance of these biomarkers. Standardized guidelines such as the mREMARK score and the use of ROC curves may help to streamline biomarker performance reporting, while external validation or at least internal cross-validation techniques may help instil confidence that biomarkers can be translated successfully into the real-world clinical realm. In future, the study of biomarkers in COPD exacerbations should not only encompass these principles, but also incorporate stringent and consistent definitions of an exacerbation. Collection of blood samples prior to the initiation of therapies and at multiple longitudinal time points following an exacerbation are also necessary to prevent possible confounding by medications and to characterize the activity of the biomarker along the time course of disease. Only then can the field progress towards a working biomarker in COPD.

## Supporting Information

S1 PRISMA Checklist PRISMA guidelines checklist. (DOC)

S1 Table List of MeSH terms used for the systematic review. (DOCX)

S2 Table Guidelines for REMARK scores. (DOCX)

S3 Table Patient characteristics of 59 publications included in the review arranged by the latest published year. (DOCX)

S4 Table COPD definitions of 59 publications included in the review arranged by the latest published year. (DOCX)

S5 Table Top three most studied AECOPD biomarkers. (DOCX)

S6 Table Biomarkers investigated in a single study. (DOCX)

S7 Table Modified REMARK (mREMARK) scores breakdown for the 12 studies listed in Table 2. (DOCX)
