# Supplementary material for: A Systematic Review of Diagnostic Biomarkers of COPD Exacerbation
Source: PLoS One. 2016 Jul 19;11(7):e0158843. doi: 10.1371/journal.pone.0158843 (PMC4951145; doi:10.1371/journal.pone.0158843)
Supplement: S1 Table — (DOCX) [file pone.0158843.s002.docx]

S1 Table: List of MeSH terms used for the systematic review

("pulmonary disease, chronic obstructive"[MeSH Terms] OR ("pulmonary"[All Fields] AND "disease"[All Fields] AND "chronic"[All Fields] AND "obstructive"[All Fields]) OR "chronic obstructive pulmonary disease"[All Fields] OR "copd"[All Fields]) AND (exacerbations[All Fields] OR “acute exacerbations”[All Fields] OR “aecopd”[All Fields]) AND ("biomarkers"[MeSH Terms] OR "biomarkers"[All Fields] OR “biological markers”[All Fields]) AND ("diagnosis"[MeSH Terms] OR "diagnosis"[All Fields] OR "diagnostic"[All Fields]) AND ("blood"[Subheading] OR "blood"[All Fields] OR "blood"[MeSH Terms] OR “serum”[All Fields] OR “plasma”[All Fields])
