# Supplementary material for: A Systematic Review of Diagnostic Biomarkers of COPD Exacerbation
Source: PLoS One. 2016 Jul 19;11(7):e0158843. doi: 10.1371/journal.pone.0158843 (PMC4951145; doi:10.1371/journal.pone.0158843)
Supplement: S3 Table — (DOCX) [file pone.0158843.s004.docx]

S3 Table. Patient characteristics of 59 publications included in the review arranged by the latest published year

| **Reference** | **Number of patients** | **Age (Mean ± SD)** | **Sex (M:F)** | **FEV1% Predicted (Mean ± SD)** | **Smoking Status** | **Smoking History in Pack-years (Mean ± SD)** |
| --- | --- | --- | --- | --- | --- | --- |
| Andelid, K., et al. [[19](#_ENREF_19)] | 60 AECOPD | 62 (45-76)* | 26:34 | 60 (29-97)* ^ | All Smokers | 40 (14-156)* |
|  | 10 Smoker Controls | 50 (26-64)* | 2:8 | 106 (83-119)* ^ | All Current Smokers | 27 (12-44)* |
|  | 10 Non-Smoker Controls | 68 (47-70)* | 3:7 | 120 (97-137)* ^ | All Non-Smokers | 0 |
| Gumus, A., et al. [[20](#_ENREF_20)] | 43 AECOPD | 68 ± 8 | 40:3 | 37 (25-49)* | 10 Current Smokers | 53 (33-73)* |
|  | 30 Controls | 64 ± 7 | 25:5 | N/A | 10 Current Smokers | 40 (23-57)* |
| Chang, C., Yao, W. [[21](#_ENREF_21)] | 57 FE COPD | 67 (63-74)* | 50:7 | 45 (36-54)* | 12 Current; 40 Ex-Smokers | 30 (20-40)* |
|  | 78 NE COPD | 66 (60-74)* | 69:9 | 52 (43-55)* | 17 Current; 50 Ex-Smokers | 20 (15-30)* |
| Chang, C., et al. [[22](#_ENREF_22)] | 93 AECOPD | 67 (61-74)* | 83:10 | 47 (43-55)* | 21 Current Smokers | 25 (22-34)* |
| Fattouh, M. Alkady, O. [[23](#_ENREF_23)] | 98 AECOPD | 62.29±7.03 | 82:16 | 53.41 ± 7.469 | 24 Current; 58 Ex; 16 Non-Smokers | N/A |
|  | 30 Controls | N/A | 23:7 | 88.4 ± 5.137 | 5 Current; 7 Ex; 17 Non-Smokers | N/A |
| Johansson, S.L., et al. [[24](#_ENREF_24)] | 14 AECOPD | 66 ± 8 | N/A | 30 ± 11 | 5 Current Smokers | 60 (34)* |
|  | 69 Stable COPD | 62 ± 7 | 32:37 | 50 ± 16 | 21 Current; 48 Ex-Smokers | 40 (20)* |
|  | 54 Smoker Controls | 46 ± 12 | N/A | N/A | All Smokers | N/A |
|  | 52 Non-smoker Controls | 41 ± 14 | N/A | N/A | All Non-Smokers | N/A |
| Labib, S., et al. [[25](#_ENREF_25)] | 20 AECOPD | 66.4 ± 11.2 | 17:3 | N/A | 9 Current; 11 Ex-Smokers | 55.6 ± 23.7 |
|  | 20 Controls | 59.3 ± 13.6 | 15:5 | N/A | N/A | 10.1 ± 4.2 |
| Lee, S.J., et al. [[26](#_ENREF_26)] | 64 AECOPD | 73.1 ± 8 | 58:6 | 52.2 ± 25.3 | 16 Current Smokers; 48 Ex-Smokers | 44.4 ± 21.0 |
|  | 68 Stable COPD | 70.3 ± 6.3 | 64:4 | 58.8 ± 20.7 | 17 Current Smokers; 51 Ex-Smokers | 38.1 ± 13.3 |
|  | 30 Controls | 70.8 ± 4.1 | 26:4 | 98.1 ± 19.5 | 3 Current Smokers; 5 Ex-Smokers | N/A |
| Liu, H.C., et al. [[27](#_ENREF_27)] | 9 AECOPD | 72 ± 4.7 | 9:0 | N/A | All Smokers | N/A |
|  | 12 Asthma | 48 ± 8.5 | 5:7 | N/A | 5 Current; 7 Non-Smokers | N/A |
|  | 10 Controls | 44 ± 8.5 | 5:5 | N/A | All Non-Smokers | N/A |
| Liu, Y., et al. [[28](#_ENREF_28)] | 27 AECOPD | 70 ± 9 | 24:3 | 34 ± 13 | All Ex-Smokers | 38.5 |
|  | 26 Stable COPD | 65 ± 8 | 24:2 | 37 ± 17 | All Ex-Smokers | 40.9 |
|  | 24 Controls | 66 ± 9 | 20:4 | 101 ± 11 | All Ex-Smokers | 39.2 |
| Meng, D.Q., et al. [[29](#_ENREF_29)] | 79 AECOPD | 67.0 ± 9.4 | 67:12 | 35.05 ± 17.98 | N/A | 30 (0–45)* |
|  | 29 Stable COPD | 64.9 ± 8.3 | 25:4 | 34.94 ± 13.18 | N/A | 42 (30–60)* |
|  | 20 Controls | 61.6 ± 9.0 | 15:5 | 103.3 ± 8.42 | N/A | 30 (6.5–34.5)* |
| Nikolakopoulou, S., et al. [[30](#_ENREF_30)] | 90 AECOPD | 69.64 ± 9.93 | 67:23 | N/A | Current or Ex-Smokers | N/A |
| Nishimura, K., et al. [[31](#_ENREF_31)] | 61 AECOPD | 75.4 ± 7.6 | 49:12 | 56.0 ± 23.8 | N/A | 76 ± 41 |
|  | 190 Stable COPD | 71.7 ± 8.7 | 166:24 | 66.6 ± 27.4 | 32 Current; 158 Ex-Smokers | 71 ± 42 |
| Omar, M.M., et al. [[32](#_ENREF_32)] | 15 Obese AECOPD | 53.13 ± 5.08 | 15:0 | N/A | All Smokers | N/A |
|  | 25 Non-Obese AECOPD | 54.88 ± 5.25 | 25:0 | N/A | All Smokers | N/A |
|  | 7 Obese Controls | 48.75 ± 5.4 | 7:0 | N/A | All Smokers | N/A |
|  | 8 Non-Obese Controls | 47.75 ± 6.43 | 8:0 | N/A | All Smokers | N/A |
| Oraby, S.S., et al. [[33](#_ENREF_33)] | 68 AECOPD | 53.43 ± 5.26 | 68:0 | 43.75 ± 0.75 | All Smokers | 30.97 ± 13.15 |
|  | 20 controls | 52.24 ± 4.86 | 20:0 | 79.46 ± 0.27 | Non Smokers | N/A |
| Urban, M.H., et al. [[34](#_ENREF_34)] | 29 AECOPD | 64 ± 7.6 | 8:21 | 37 ± 12 | N/A | 69 ± 46 |
| Zhang, Y., et al. [[35](#_ENREF_35)] | 44 AECOPD | 68.2 ± 8.5 | 32:12 | 47.5 ± 13.5 | 19 Current; 11 Ex; 14 Non-Smokers | 39.5 ± 17.6 |
| Zhao, Y.F., et al. [[36](#_ENREF_36)] | 78 AECOPD | 74 ± 12 | 53:25 | 40.87 ± 14.16 | N/A | 47 (35–68)* |
|  | 81 Stable COPD | 70 ± 19 | 59:22 | 57.04 ± 19.74 | N/A | 45 (32–60)* |
| Adnan, A.M., et al. [[37](#_ENREF_37)] | 35 AECOPD | 65.37 | 74:14 | N/A |  | N/A |
|  | 30 Stable COPD | 63.36 |  | N/A |  | N/A |
|  | 23 Controls | 62.78 |  | N/A | All Non-Smokers | N/A |
| Carter, R.I., et al. [[38](#_ENREF_38)] | 81 AECOPD | 65.75 ± 0.92# | 45:36 | 73.28 ± 2.86# | All Smokers or ex-smokers | N/A |
| Gao, P., et al. [[39](#_ENREF_39)] | 83 AECOPD | 63.23 ± 11.42 | 61:22 | 39.8 ± 14.7 | 40 Current; 43 Non-Smokers | 19.11 ± 11.92 |
|  | 26 Controls | 60.44 ± 13.42 | 25:1 | 93.0 ± 14.7 | 9 Current; 17 Non-Smokers | 15.32 ± 13.85 |
| Jin, Q., et al. [[40](#_ENREF_40)] | 100 AECOPD | 76 (66-91)$ | 72:28 | 40 (24–66)$ | N/A | N/A |
|  | 46 Stable COPD | 75 (65–89)$ | 34:12 | N/A | N/A | N/A |
|  | 50 Controls | 75 (65–86)$ | 34:16 | N/A | N/A | N/A |
| Mohamed, N.A., et al. [[41](#_ENREF_41)] | 40 AECOPD | 68.32 ± 6.6 | N/A | 55.28 ± 24 | N/A | N/A |
|  | 20 Stable COPD | 64.4 ± 5.6 | N/A | 71.52 ± 22 | N/A | N/A |
|  | 20 Controls | 62.72 ± 4.2 | N/A | N/A | N/A | N/A |
| Patel, A.R.C., et al. [[42](#_ENREF_42)] | 55 AECOPD | 72.1 ± 8.4 | 32:23 | 46.7 ± 18.5 | 11 Current Smokers | 44 (21-74)* |
|  | 98 Stable COPD | 72.1 ± 8.9 | 60:38 | 52.0 ± 18.9 | 20 Current Smokers | 45 (25-79)* |
| Scherr, A., et al. [[43](#_ENREF_43)] | 200 AECOPD | 70 (42-91)$ | 114:86 | 40 ± 18.3 | N/A | 45 ± 28 |
|  | 133 Stable COPD | 64.9 ± 12.3 | 99:34 | 47 ± 17 | N/A | N/A |
|  | 40 Controls | 59.2 ± 7 | 22:18 | N/A | N/A | N/A |
| Shoukry, A., et al. [[44](#_ENREF_44)] | 20 AECOPD | 56.8 ± 8.3 | 18:2 | 44.6 ± 11.9 | All male ex-smokers; females non-smokers | N/A |
|  | 40 Stable COPD | 54.5 ± 7.6 | 35:5 | 54.8 ± 14.5 | All male ex-smokers; females non-smokers | N/A |
|  | 20 Controls | 53.6 ± 8.2 | 17:3 | 94.7 ± 12.6 | All Non-Smokers | N/A |
| Stanojkovic, I., et al. [[45](#_ENREF_45)] | 85 AECOPD | 61.9 ± 7.9 | 34:51 | 41.9 ± 17.4 AECOPD | 28 Current; 41 Ex; 16 Non-Smokers | N/A |
|  |  |  |  | 57.9 ± 25.3 Stable COPD |  |  |
|  | 47 Controls | 59.1 ± 4.4 | 22:25 | N/A | 14 Current; 21 Ex; 12 Non-Smokers | N/A |
| Chen, H., et al. [[46](#_ENREF_46)] | 6 AECOPD | 69 ± 10.35# | 3:3 | 55.72 ± 12.57# | 3 Current; 3 Non-Smokers | N/A |
|  | 6 Stable COPD | 61.50 ± 8.43# | 3:3 | 64.72 ± 15.23# | 3 Current; 3 Non-Smokers | N/A |
|  | 6 Controls | 61.17 ± 9.16# | 3:3 | 84.72 ± 10.57# | 3 Current; 3 Non-Smokers | N/A |
| Falsey, A.R., et al. [[47](#_ENREF_47)] | 184 AECOPD | 66.7 ± 13.3 | 97:87 | N/A | N/A | N/A |
| Huang, J., et al. [[48](#_ENREF_48)] | 102 AECOPD | 72 (66-79)* | 43:59 | 47 ± 18 | 55 Current; 33 Ex-Smokers | ≥ 10 |
|  | 53 Stable COPD | 65 ± 7 | 23:30 | 62 (44-77)* | 28 Current: 25 Ex-Smokers | ≥ 10 |
|  | 26 Controls (Non-Smokers) | 51 ± 13 | 17:29 | 106 (95-115)* | All Non-Smokers | N/A |
|  | 20 Controls  (Smokers) | 52 ± 9 | 9:11 | 93 (88-100)* | All Smokers | N/A |
|  | 19 Controls (group 2) | 68 (65-73)* | 18:1 | N/A | 10 Current; 9 Non-Smokers | N/A |

| Ju, C.R., et al. [[49](#_ENREF_49)] | 40 AECOPD | 66.05 ± 5.67 | 38:2 | 33.78 ± 7.39 | All Ex-Smokers | N/A |
| --- | --- | --- | --- | --- | --- | --- |
|  | 71 Stable COPD | 65.17 ± 6.80 | 54:17 | 37.76 ± 14.93 | All Ex-Smokers | N/A |
|  | 60 Controls | 63.98 ± 5.77 | 21:39 | 97.1 ± 8.90 | All Non-Smokers | N/A |
| Koczulla, A.R., et al. [[50](#_ENREF_50)] | 18 AECOPD | 72.2 ± 11.8 | 13:5 | 59.2 ± 14.5 | 1 Current; 17 Ex-Smokers | 43.9 ± 26.4 |
|  | 17 Stable COPD | 66.6 ± 7.8 | 11:6 | 56.3 ± 17.5 | 3 Current; 14 Ex-Smokers | 38.8 ± 21.5 |
|  | 10 Controls | 36.1 ± 11.5 | 6:4 | 103.5 ± 6.8 | 3 Ex-Smokers | 4 ± 7.4 |
| Kwiatkowska, S., et al. [[51](#_ENREF_51)] | 17 AECOPD | 68 ± 10 | 10:7 | 57 ± 15 | All Current Smokers | 33 ± 19 |
|  | 22 Controls | 57 ± 11 | 14:8 | 95 ± 12 | All Current Smokers | 28 ± 11 |
| Marcun, R., et al. [[52](#_ENREF_52)] | 127 AECOPD | 70 ± 10 | 89:38 | 34 ± 15 | 28 Current; 99 Ex-Smokers | 48 ± 30 |
| Mohamed, K.H., et al. [[53](#_ENREF_53)] | 50 AECOPD | 64.6 ± 8.0 | 37:13 | 53.2 ± 9.5 | 42 Current Smokers | 40.4 ± 8.9 |
|  | 10 Controls | 56.3 ± 11.5 | 8:2 | 88 ± 10.4 | 6 Current Smokers | 28 ± 6 |
| Pazarli, A.C., et al. [[54](#_ENREF_54)] | 68 AECOPD | 65.9 ± 0.97# | 58:10 | N/A | N/A | 46.1 ± 2.89# |
|  | 50 Stable COPD | 64.1 ± 1.22# | 46:4 | N/A | N/A | 45 ± 2.88# |
| Rohde, G., et al. [[55](#_ENREF_55)] | 118 AECOPD | 66 (13)* | 95:23 | 35.1 (20.9)* | N/A | 30 (32)* |
|  | 64 Stable COPD | 67 (19)* | 56:8 | 45.4 (29.6)* | N/A | 30 (47)* |
|  | 13 Smoker Controls | 47.5 (4)* | 7:6 | 98.4 (13.5)* | N/A | 40.5 (26.8)* |

| Shaker, A., et al. [[56](#_ENREF_56)] | 20 AECOPD | 57.34 ± 12.65 | 20:0 | 46.39 ± 12.15 | N/A | 48.57 ± 15.37 |
| --- | --- | --- | --- | --- | --- | --- |
|  | 10 Controls | 55.58 ± 13.21 | 10:0 | 91.23 ± 14.46 | N/A | 46.39 ± 13.46 |
| Yerkovich, S.T., et al. [[57](#_ENREF_57)] | 32 AECOPD | 69.6 (62.8-74.2)* | 21:11 | 31 (21-45) | 9 Current Smokers | 45 (35-72)* |
|  | 28 COPD | 69.8 (62.9-73.4)* | 15:13 | 46 (35-60) | 8 Current Smokers | 53 (37-72)* |
| Bafadhel, M., et al. [[13](#_ENREF_13)] | 145 COPD | 69 (43-88)$ | 101:44 | 52 ± 2# | 42 Current; 100 Ex-Smokers | 49 (10-153)$ |
| Chen, H., et al. [[58](#_ENREF_58)] | 7 AECOPD | 83 ± 7 | 4:3 | N/A | N/A | N/A |
|  | 5 Stable COPD | N/A | N/A | N/A | N/A | N/A |
|  | 5 Controls | N/A | N/A | N/A | N/A | N/A |
| Lacoma, A., et al. [[59](#_ENREF_59)] | 217 AECOPD | 71.38 ± 9.97 | 204:13 | 102 <40%; 51 40%-59%; 17 60%-80% 47 N/A | 38 Current; 150 Ex; 23 Non-Smokers; 6 N/A | N/A |
|  | 46 Stable COPD | 70.93 ± 10.37 | 45:1 | 11 <40%; 22 40%-59%; 3 60%-80%; 10 N/A | 15 Current; 23 Ex-Smokers; 8 N/A | N/A |
| Lacoma, A., et al. [[60](#_ENREF_60)] | 217 AECOPD | 71.4 ± 9.9 | 204:13 | 102 <40%; 51 40%-59%; 17 60%-80% 47 N/A | 38 Current; 150 Ex; 23 Non-Smokers; 6 N/A | N/A |
|  | 46 Stable COPD | 70.9 ± 10.3 | 45:1 | 11 <40%; 22 40%-59%; 3 60%-80%; 10 N/A | 15 Current; 23 Ex-Smokers; 8 N/A | N/A |

| Lim, S.C., et al. [[61](#_ENREF_61)] | 17 AECOPD | 68.3 ± 7.7 | 13:4 | 36.7 ± 14.5 | 9 Current Smokers | 35.2 ± 23.7 |
| --- | --- | --- | --- | --- | --- | --- |
|  | 21 Stable COPD | 64.9 ± 7.1 | 20:1 | 56.9 ± 15.6 | 11 Current Smokers | 36.1 ± 19.2 |
|  | 12 Controls | 62.5 ± 7.9 | 11:1 | 103.0 ± 11.5 | 7 Current Smokers | 33.3 ± 17.2 |
| Markoulaki, D., et al. [[62](#_ENREF_62)] | 93 AECOPD | 66 ± 9 | 64:29 | 45 (34–64)* | 52 Current; 41 Ex-Smokers | 85 ± 45 |
| Krommidas, G., et al. [[63](#_ENREF_63)] | 63 AECOPD | 67.4 ± 9.1 | 54:9 | 42.8 ± 13.4 | 38 Current; 25 Ex-Smokers | 92.5 ± 49.8 |
| Quint, J.K., et al. [[64](#_ENREF_64)] | 136 AECOPD | 72.6 ± 8.4 | 83:53 | 53.9 ± 18.7 | 41 Current Smokers | 51.1 ± 38.6 |
|  | 70 Controls | 67.4 ± 8.7 | 28:42 | 112.1 ± 28.3 | 12 Current Smokers | 18.4 ± 20.9 |
| Koutsokera, A., et al. [[65](#_ENREF_65)] | 30 AECOPD | 69.3 ± 1.7# | 28:2 | 38.6 ± 3.5# | 14 Current; 16 Ex-Smokers | 89.7 ± 10.0# |
| Kythreotis, P., et al. [[66](#_ENREF_66)] | 52 AECOPD | 65.8 ± 8.3 | 43:9 | 44.1 ± 11.4 | All current or ex-smokers | N/A |
|  | 25 Controls | 65.9 ± 9.6 | 19:6 | 89.9 ± 8.0 | N/A | N/A |
| Shakoori, T.A., et al. [[67](#_ENREF_67)] | 13 AECOPD | 60 ± 13 | 13:0 | 45 ± 21 | 9 Current; 4 Ex-Smokers | 53 ± 23 |
|  | 14 Stable COPD | 62 ± 11 | 14:0 | 50 ± 24 | 8 Current; 6 Ex-Smokers | 75 ± 41 |
|  | 54 Controls | 36 ± 11 | 54:0 | 98 ± 13 | 35 Current; 5 Ex; 13 Non-Smokers | 16 ± 20 |
| Karadag, F., et al. [[68](#_ENREF_68)] | 20 AECOPD | 68.60 ± 5.87 | 20:0 | 36.00 ± 9.92 | N/A | 61.50 ± 22.05 |
|  | 83 Stable COPD | 65.54 ± 7.66 | 83:0 | 46.41 ± 14.43 | N/A | 53.58 ± 25.63 |
|  | 30 Controls | 64.10 ± 7.68 | 30:0 | 85.09 ± 10.24 | N/A | 16.77 ± 15.78 |

| Stolz, D., et al. [[69](#_ENREF_69)] | 208 AECOPD | 70.3 ± 9.9 | 94:114 | 41 ± 17 | 94 Current; 97 Ex-Smokers | 45 ± 27.9 |
| --- | --- | --- | --- | --- | --- | --- |
| Groenewegen, K.H., et al. [[70](#_ENREF_70)] | 21 AECOPD | 66.7 ± 9.0 | 15:6 | 35.0 ± 14.4 | 7 Current; 13 Ex; 1 Non-Smokers | 40 ± 20 |
|  | 20 Controls | 60.6 ± 3.4 | 14:6 | 108.2 ± 14.2 | 1 Current; 11 Ex; 8 Non-Smokers | 20 ± 15 |
| Perera, W. R., et al. [[71](#_ENREF_71)] | 73 AECOPD | 69.3 ± 7.8 | 39:34 | 45 ± 18 | 20 Current Smokers | 48.1 ± 34.9 |
| Pinto-Plata, V. M., et al. [[72](#_ENREF_72)] | 20 AECOPD | 72 ± 8 | N/A | 41 ± 13 | All Ex-Smokers | 68 ± 27 |
| Hurst, J.R., et al. [[73](#_ENREF_73)] | 90 AECOPD | 70.1 ± 8.2 | 54:36 | 43.9 (27.5–56.8)* | 25 Current Smokers | 45 (29–59)* |
| Phua, J., et al. [[74](#_ENREF_74)] | 43 COPD | 72 ± 14 | 37:6 | N/A | N/A | 50 (0-180) |
|  | 63 Controls | 33 ± 11 | 43:20 | N/A | N/A | N/A |
| Roland, M., et al. [[75](#_ENREF_75)] | 71 AECOPD | 68.2 ± 7.8 | N/A | 39.8 ± 17.0 | 26 Current Smokers | 44.6 ± 34.1 |
| Fiorini, G., et al. [[76](#_ENREF_76)] | 17 AECOPD | 69.5 ± 2 | 13:4 | N/A | 10 Current Smokers | 1 pack per day |

Numerical results displayed as Mean ± SD unless otherwise indicated by symbols. Symbols: N/A: Not available, *: Median (IQR), #: Mean ± SEM, and $: Mean (Range), ^: Without the use of bronchodilation prior to lung measurement. Abbreviations: AECOPD = acute exacerbation of COPD group, FE = frequent exacerbators, and NE = non-frequent exacerbators.
