# Supplementary material for: A Systematic Review of Diagnostic Biomarkers of COPD Exacerbation
Source: PLoS One. 2016 Jul 19;11(7):e0158843. doi: 10.1371/journal.pone.0158843 (PMC4951145; doi:10.1371/journal.pone.0158843)
Supplement: S4 Table — (DOCX) [file pone.0158843.s005.docx]

**S4 Table. COPD definitions of 59 publications included in the review arranged by the latest published year**

| **Reference** | **COPD Diagnosis** | **AECOPD Definition** | **Stable COPD Definition** |
| --- | --- | --- | --- |
| Andelid, K., et al. [[19](#_ENREF_19)] | GOLD criteria | Infections in airways or lungs were deemed to be exacerbations | 4 weeks free from exacerbation |
| Gumus, A., et al. [[20](#_ENREF_20)] | GOLD criteria | W | N/A |
| Chang, C. , Yao, W. [[21](#_ENREF_21)] | GOLD criteria | W | 8 weeks free from exacerbation |
| Chang, C., et al. [[22](#_ENREF_22)] | GOLD criteria | W | 8 weeks free from exacerbation |
| Fattouh, M. Alkady, O. [[23](#_ENREF_23)] | GOLD criteria | N/A | 4 weeks free from exacerbation |
| Johansson, S.L., et al. [[24](#_ENREF_24)] | GOLD criteria | N/A | 4 weeks free from exacerbation |
| Labib, S., et al. [[25](#_ENREF_25)] | GOLD criteria | BAP-65 Score | One month after exacerbation |
| Lee, S.J., et al. [[26](#_ENREF_26)] | GOLD criteria | W | 3 months free from exacerbation |
| Liu, H.C., et al. [[27](#_ENREF_27)] | GOLD criteria | W | N/A |
| Liu, Y., et al. [[28](#_ENREF_28)] | GOLD criteria | W | No requirement for increased treatment for 12 weeks; GOLD II to IV |
| Meng, D.Q., et al. [[29](#_ENREF_29)] | GOLD criteria | W | No requirement for increased treatment for 30 days |
| Nikolakopoulou, S., et al. [[30](#_ENREF_30)] | GOLD criteria | W | 8 weeks free from exacerbation |
| Nishimura, K., et al. [[31](#_ENREF_31)] | GOLD criteria | W | 4 weeks free from exacerbation |
| Omar, M.M., et al. [[32](#_ENREF_32)] | GOLD criteria | W | Disappearance of symptoms |
| Oraby, S.S., et al. [[33](#_ENREF_33)] | GOLD criteria | W | No requirement for increased treatment for 12 weeks |
| Urban, M.H., et al. [[34](#_ENREF_34)] | GOLD criteria | W | 8 weeks free from exacerbation |
| Zhang, Y., et al. [[35](#_ENREF_35)] | N/A | W | Disappearance of symptoms |
| Zhao, Y.F., et al. [[36](#_ENREF_36)] | GOLD criteria | W | N/A |
| Adnan, A.M., et al. [[37](#_ENREF_37)] | N/A | N/A | 8 weeks free from exacerbation |
| Carter, R.I., et al. [[38](#_ENREF_38)] | History of chronic bronchitis | Anthonisen criteria | 8 weeks after exacerbation |
| Gao, P., et al. [[39](#_ENREF_39)] | GOLD criteria | N/A | N/A |
| Jin, Q., et al. [[40](#_ENREF_40)] | GOLD criteria | W & ICU criteria | 4 weeks free from exacerbation |
| Mohamed, N.A., et al. [[41](#_ENREF_41)] | GOLD criteria | W | 3 months free from exacerbation |
| Patel, A.R.C., et al. [[42](#_ENREF_42)] | GOLD criteria | W | 4 weeks free from exacerbation |
| Scherr, A., et al. [[43](#_ENREF_43)] | GOLD criteria | Anthonisen criteria | N/A |
| Shoukry, A., et al. [[44](#_ENREF_44)] | GOLD criteria | W | 3 months free from exacerbation |
| Stanojkovic, I., et al. [[45](#_ENREF_45)] | GOLD criteria | W | 30 days after exacerbation |
| Chen, H., et al. [[46](#_ENREF_46)] | GOLD criteria | W | No requirement for increased treatment for 30 days; 8 weeks free from exacerbation |
| Falsey, A.R., et al. [[47](#_ENREF_47)] | GOLD criteria | Anthonisen criteria | N/A |
| Huang, J., et al. [[48](#_ENREF_48)] | GOLD criteria | Symptoms of cough and breathlessness | 4 weeks free from exacerbation & 2 weeks free from chest infections |
| Ju, C.R., et al. [[49](#_ENREF_49)] | GOLD criteria | W | 3 months free from exacerbation |
| Koczulla, A.R., et al. [[50](#_ENREF_50)] | GOLD criteria | W | Free of serum signs of inflammation and radiological signs of pneumonia |
| Kwiatkowska, S., et al. [[51](#_ENREF_51)] | GOLD criteria | W | N/A |
| Marcun, R., et al. [[52](#_ENREF_52)] | GOLD criteria | W | N/A |
| Mohamed, K.H., et al. [[53](#_ENREF_53)] | GOLD criteria | W | N/A |
| Pazarli, A.C., et al. [[54](#_ENREF_54)] | GOLD criteria | Anthonisen criteria | 4 weeks free from exacerbation |
| Rohde, G., et al. [[55](#_ENREF_55)] | GOLD criteria | Anthonisen criteria | 4 weeks free from exacerbation; no requirement for increased treatment for 14 days |
| Shaker, A., et al. [[56](#_ENREF_56)] | GOLD criteria | W | One month after discharge |
| Yerkovich, S.T., et al. [[57](#_ENREF_57)] | GOLD criteria | W | 6 weeks free from exacerbation |
| Bafadhel, M., et al. [[13](#_ENREF_13)] | GOLD criteria | Anthonisen criteria | 8 weeks free from exacerbation |
| Chen, H., et al. [[58](#_ENREF_58)] | N/A | W | No requirement for increased treatment for 30 days |
| Lacoma, A., et al. [[59](#_ENREF_59)] | SEPAR guidelines | Anthonisen criteria | 4 weeks free from exacerbation |
| Lacoma, A., et al. [[60](#_ENREF_60)] | SEPAR guidelines | Anthonisen criteria | 4 weeks free from exacerbation |
| Lim, S.C., et al. [[61](#_ENREF_61)] | GOLD criteria | Anthonisen criteria | 8 weeks free from exacerbation |
| Markoulaki, D., et al. [[62](#_ENREF_62)] | GOLD criteria | Type 1 Anthonisen criteria only | 8 weeks free from exacerbation & respiratory tract infection |
| Krommidas, G., et al. [[63](#_ENREF_63)] | GOLD criteria | Type 1 Anthonisen criteria only | 8 weeks free from exacerbation & respiratory tract infection |
| Quint, J.K., et al. [[64](#_ENREF_64)] | GOLD criteria | W | > 42 days post-exacerbation and > 14 days pre-exacerbation onset |
| Koutsokera, A., et al. [[65](#_ENREF_65)] | GOLD criteria | Type 1 Anthonisen criteria only | 10 or 40 days after exacerbation |
| Kythreotis, P., et al. [[66](#_ENREF_66)] | GOLD criteria | W | 3 months free from exacerbation |
| Shakoori, T.A., et al. [[67](#_ENREF_67)] | GOLD criteria | W | N/A |
| Karadag, F., et al. [[68](#_ENREF_68)] | GOLD criteria | Anthonisen criteria | 3 months free from exacerbation |
| Stolz, D., et al. [[69](#_ENREF_69)] | GOLD criteria | Anthonisen criteria | 14 to 18 days after exacerbation |
| Groenewegen, K.H., et al. [[70](#_ENREF_70)] | GOLD criteria | Type 1 Anthonisen criteria only | 6 months after discharge |
| Perera, W. R., et al. [[71](#_ENREF_71)] | GOLD criteria | W | 6 weeks free from exacerbation |
| Pinto-Plata, V.M., et al. [[72](#_ENREF_72)] | GOLD criteria | Anthonisen criteria | 8 weeks after discharge |
| Hurst, J.R., et al. [[73](#_ENREF_73)] | GOLD criteria | W | 4 weeks free from exacerbation |
| Phua, J., et al. [[74](#_ENREF_74)] | GOLD criteria | Anthonisen criteria | N/A |
| Roland, M., et al. [[75](#_ENREF_75)] | GOLD criteria | Anthonisen criteria | 3 weeks free from exacerbation |
| Fiorini, G., et al. [[76](#_ENREF_76)] | GOLD criteria | Exacerbation requiring hospital admission | N/A |

Anthonisen criteria were based on patients evaluated for dyspnea, sputum production or increased purulence, without radiological consolidation. Anthonisen type 1 criteria defined patients that were presented with all three symptoms of increased dyspnea, sputum volume and sputum purulence. BAP-65 score definition included BUN level >25 mg/dl, altered sensorium, and pulse rate >109beats/min, and age > 65. Abbreviations: AECOPD = acute exacerbation of chronic obstructive pulmonary disease, GOLD = Global Initiative for chronic obstructive lung disease, SEPAR = Spanish Society of Pneumology and Thoracic Surgery, W = a sustained worsening of the patient’s condition of dyspnea sensation, coughing, or sputum production that can become purulent, from the stable state and beyond normal day-to-day variations, necessitating a change in regular medication in a patient with underlying COPD.
