# Supplementary material for: A Systematic Review of Diagnostic Biomarkers of COPD Exacerbation
Source: PLoS One. 2016 Jul 19;11(7):e0158843. doi: 10.1371/journal.pone.0158843 (PMC4951145; doi:10.1371/journal.pone.0158843)
Supplement: S5 Table — (DOCX) [file pone.0158843.s006.docx]

S5 Table. Top three most studied AECOPD biomarkers

| **Reference** | **Year** | **Number of patients** | **CRP** | **IL-6** | **TNF-α** | **Assay methodology** |
| --- | --- | --- | --- | --- | --- | --- |
| Andelid, K., et al. [[19](#_ENREF_19)] | 2015 | 60 AECOPD | ^ + |  |  | N/A |
| Gumus, A., et al. [[20](#_ENREF_20)] | 2015 | 43 AECOPD | ↑ +, ^ + |  |  | N/A |
| Chang, C. , Yao, W. [[21](#_ENREF_21)] | 2014 | 135 AECOPD | ↑ + | ↑ |  | Latex agglutination assay, ELISA |
| Chang, C., et al. [[22](#_ENREF_22)] | 2014 | 93 AECOPD | ↑ + | ↑ + |  | Latex agglutination assay, ELISA |
| Fattouh, M. Alkady, O. [[23](#_ENREF_23)] | 2014 | 98 AECOPD | ↑ +, ^ + |  |  | Latex agglutination assay |
| Johansson, S.L., et al. [[24](#_ENREF_24)] | 2014 | 14 AECOPD : 69 COPD | ↑ |  |  | N/A |
| Liu, Y., et al. [[28](#_ENREF_28)] | 2014 | 27 AECOPD : 26 COPD | ↑ +, ^ + |  |  | Immunoturbidimetric assay |
| Meng, D.Q., et al. [[29](#_ENREF_29)] | 2014 | 79 AECOPD : 29 COPD | NP |  |  | N/A |
| Nikolakopoulou, S., et al. [[30](#_ENREF_30)] | 2014 | 90 AECOPD | ↑ + |  |  | Immunoturbidimetric assay |
| Zhang, Y., et al. [[35](#_ENREF_35)] | 2014 | 44 AECOPD | ↑ + |  |  | Hospital analyzer |
| Zhao, Y.F., et al. [[36](#_ENREF_36)] | 2014 | 78 AECOPD : 81 COPD | ↑ + |  |  | Immunonephelometric assay |
| Gao, P., et al. [[39](#_ENREF_39)] | 2013 | 83 AECOPD | ^ + | ^ + |  | ELISA |
| Mohamed, N.A., et al. [[41](#_ENREF_41)] | 2013 | 40 AECOPD : 20 COPD | ↑ + | ↑ +, ^ + | ↑ +, ^ + | Immunonephelometric assay, ELISA |
| Patel, A.R.C., et al. [[42](#_ENREF_42)] | 2013 | 55 AECOPD : 98 COPD | ↑ + |  |  | Electro-chemiluminescence immune assay |
| Shoukry, A., et al. [[44](#_ENREF_44)] | 2013 | 20 AECOPD : 40 COPD |  | ↑ −, ^ + | ↑ +, ^ + | ELISA |
| Stanojkovic, I., et al. [[45](#_ENREF_45)] | 2013 | 85 AECOPD | ↑ + |  |  | Immunoturbidimetric assay |
| Ju, C.R., et al. [[49](#_ENREF_49)] | 2012 | 40 AECOPD : 71 COPD | ↑ +, ^ + |  |  | ELISA |
| Koczulla, A.R., et al. [[50](#_ENREF_50)] | 2012 | 18 AECOPD : 17 COPD | ↑ +, ^ + |  |  | N/A |
| Mohamed, K.H., et al. [[53](#_ENREF_53)] | 2012 | 50 AECOPD | ↑ + |  |  | Hospital analyzer |
| Pazarli, A.C., et al. [[54](#_ENREF_54)] | 2012 | 68 AECOPD : 50 COPD | ^ + |  |  | N/A |
| Bafadhel, M., et al. [[13](#_ENREF_13)] | 2011 | 145 COPD | ↑ + | ↑ + |  | Immunoturbidimetric assay, Multiplex immuno assay |
| Chen, H., et al. [[58](#_ENREF_58)] | 2011 | 7 AECOPD : 5 COPD | NP |  |  | N/A |
| Lacoma, A., et al. [[60](#_ENREF_60)] | 2011 | 217 AECOPD : 46 COPD | ↑ + |  |  | Immunofluorescent assay |
| Lim, S.C., et al. [[61](#_ENREF_61)] | 2011 | 17 AECOPD : 21 COPD |  | ↑ +, ^ + | ↑ +, ^ + | ELISA |
| Markoulaki, D., et al. [[62](#_ENREF_62)] | 2011 | 93 AECOPD | ↑ + | ↑ + | ↑ + | Immunonephelometric assay, ELISA |
| Krommidas, G., et al. [[63](#_ENREF_63)] | 2010 | 63 AECOPD | ↑ + | ↑ + | ↑ + | Immunonephelometric assay, ELISA |
| Quint, J.K., et al. [[64](#_ENREF_64)] | 2010 | 136 AECOPD | ↑ + | ↑ + |  | Luminometric assay, ELISA |
| Koutsokera, A., et al. [[65](#_ENREF_65)] | 2009 | 30 AECOPD | ↑ + | ↑ + | ↑ + | Immunonephelometric assay, ELISA |
| Kythreotis, P., et al. [[66](#_ENREF_66)] | 2009 | 52 AECOPD |  | ↑ + | ↑ + | ELISA |
| Karadag, F., et al. [[68](#_ENREF_68)] | 2008 | 20 AECOPD : 83 COPD |  | ↑ −, ^ + | ↑ ±, ^ + | ELISA |
| Stolz, D., et al. [[69](#_ENREF_69)] | 2008 | 208 AECOPD |  | NP |  | EMIT |
| Groenewegen, K.H., et al. [[70](#_ENREF_70)] | 2007 | 21 AECOPD |  | ↑ + |  | ELISA |
| Perera, W. R., et al. [[71](#_ENREF_71)] | 2007 | 73 AECOPD | ↑ + | ↑ + |  | ELISA |
| Pinto-Plata, V. M., et al. [[72](#_ENREF_72)] | 2007 | 20 AECOPD |  | ↑ + | ↑ − | ELISA |
| Hurst, J.R., et al. [[73](#_ENREF_73)] | 2006 | 90 AECOPD | ↑ + | ↑ + | ↑ − | Chemiluminescent proteome array |

Abbreviations: ↑ = biomarker increased during AECOPD, ^ = biomarker increased during AECOPD compared to healthy controls, + = Statistically significant ( P-value < 0.05), − = Not statistically significant (P-value > 0.20), ± = Borderline statistically significant ( P-value = 0.05-0.20), NP = studied biomarker but statistically analysis not performed or not published, : = independent group comparison between exacerbating COPD patients versus stable COPD patients, ELISA = Enzyme-linked immunosorbent assay, EMIT = Enzyme multiplied immunoassay technique, N/A = Not available or not specified.
