# Supplementary material for: A Systematic Review of Diagnostic Biomarkers of COPD Exacerbation
Source: PLoS One. 2016 Jul 19;11(7):e0158843. doi: 10.1371/journal.pone.0158843 (PMC4951145; doi:10.1371/journal.pone.0158843)
Supplement: S6 Table — (DOCX) [file pone.0158843.s007.docx]

S6 Table. Biomarkers investigated in a single study

| **Biomarkers** | **Reference** | **Year** | **Direction during AECOPD** | **Statistical Significance** |
| --- | --- | --- | --- | --- |
| Adrenomedullin | Meng, D.Q., et al. [[29](#_ENREF_29)] | 2014 | ↑ | + |
| Alpha-1 antitrypsin | Koczulla, A.R., et al. [[50](#_ENREF_50)] | 2012 | ↑ | + |
| Amphiregulin | Hurst, J.R., et al. [[73](#_ENREF_73)] | 2006 | ↔ | − |
| Angiopoietin-2 | Nikolakopoulou, S., et al. [[30](#_ENREF_30)] | 2014 | ↑ | + |
| Anti-VP1 IgG1 | Yerkovich, S.T., et al. [[57](#_ENREF_57)] | 2012 | ↓ | + |
| Aα-Val360 | Carter, R.I., et al. [[38](#_ENREF_38)] | 2013 | ↑ | + |
| Beta-crosslaps | Stanojkovic, I., et al. [[45](#_ENREF_45)] | 2013 | ↑ | + |
| BPI | Groenewegen, K.H., et al. [[70](#_ENREF_70)] | 2007 | ↑ | + |
| CCL3 | Bafadhel, M., et al. [[13](#_ENREF_13)] | 2011 | ↓ | + |
| CCL13 | Bafadhel, M., et al. [[13](#_ENREF_13)] | 2011 | ↓ | + |
| CCL25 | Chen, H., et al. [[58](#_ENREF_58)] | 2011 | ↓ | − |
| CCL27 | Chen, H., et al. [[58](#_ENREF_58)] | 2011 | ↓ | − |
| CD34+ cells | Liu, Y., et al. [[28](#_ENREF_28)] | 2014 | ↓ | − |
| Cerberus 1 | Chen, H., et al. [[46](#_ENREF_46)] | 2012 | ↓ | − |
| Copeptin | Zhao, Y.F., et al. [[36](#_ENREF_36)] | 2014 | ↑ | + |
| Endothelin-1 | Roland, M., et al. [[75](#_ENREF_75)] | 2001 | ↑ | ± |
| Eotaxin/CCL11 | Adnan, A.M., et al. [[37](#_ENREF_37)] | 2013 | ↑ | + |
| Eotaxin-2/CCL24 | Hurst, J.R., et al. [[73](#_ENREF_73)] | 2006 | ↓ | − |
| EPO | Markoulaki, D., et al. [[62](#_ENREF_62)] | 2011 | ↑ | + |
| Fibronectin | Hurst, J.R., et al. [[73](#_ENREF_73)] | 2006 | ↑ | − |
| FSH | Shaker, A., et al. [[56](#_ENREF_56)] | 2012 | ↑ | + |
| Growth hormone R | Chen, H., et al. [[46](#_ENREF_46)] | 2012 | ↓ | − |
| Hemoglobin | Markoulaki, D., et al. [[62](#_ENREF_62)] | 2011 | ↓ | + |
| HMGB1 | Zhang, Y., et al. [[35](#_ENREF_35)] | 2014 | ↑ | + |
| IgE | Fiorini, G., et al. [[76](#_ENREF_76)] | 2000 | ↑ | − |
| IL-9 | Chen, H., et al. [[58](#_ENREF_58)] | 2011 | ↓ | − |
| IL-12 p40 | Hurst, J.R., et al. [[73](#_ENREF_73)] | 2006 | ↑ | − |
| IL-13 | Bafadhel, M., et al. [[13](#_ENREF_13)] | 2011 | ↓ | + |
| IL-19 | Chen, H., et al. [[46](#_ENREF_46)] | 2012 | ↓ | + |
| LH | Shaker, A., et al. [[56](#_ENREF_56)] | 2012 | ↑ | + |
| LTB4 | Pinto-Plata, V.M., et al. [[72](#_ENREF_72)] | 2007 | ↑ | + |
| Lymphotoxin beta | Chen, H., et al. [[46](#_ENREF_46)] | 2012 | ↓ | − |
| MFAP4 | Johansson, S.L., et al. [[24](#_ENREF_24)] | 2014 | ↓ | + |
| MMP-10 | Chen, H., et al. [[46](#_ENREF_46)] | 2012 | ↓ | − |
| MR-proANP | Lacoma, A., et al. [[59](#_ENREF_59)] | 2011 | ↑ | + |
| Neutrophil elastase | Andelid, K., et al. [[19](#_ENREF_19)] | 2015 | ↑ | + |
| NO | Karadag, F., et al. [[68](#_ENREF_68)] | 2008 | ↑ | + |
| Pancreatic stone protein/regenerating protein | Scherr, A., et al. [[43](#_ENREF_43)] | 2013 | ↑ | + |
| RBP4 | Jin, Q., et al. [[40](#_ENREF_40)] | 2013 | ↓ | + |
| sICAM-1 | Hurst, J.R., et al. [[73](#_ENREF_73)] | 2006 | ↑ | + |
| sTNFR55 | Groenewegen, K.H., et al. [[70](#_ENREF_70)] | 2007 | ↑ | − |
| sTNFR75 | Groenewegen, K.H., et al. [[70](#_ENREF_70)] | 2007 | ↑ | + |
| suPAR | Gumus, A., et al. [[20](#_ENREF_20)] | 2015 | ↑ | + |
| TEAC | Groenewegen, K.H., et al. [[70](#_ENREF_70)] | 2007 | ↓ | + |
| Testosterone | Shaker, A., et al. [[56](#_ENREF_56)] | 2012 | ↓ | + |
| Thrombopoietin | Chen, H., et al. [[46](#_ENREF_46)] | 2012 | ↓ | − |
| T3 | Shoukry, A., et al. [[44](#_ENREF_44)] | 2013 | ↓ | + |
| T4 | Shoukry, A., et al. [[44](#_ENREF_44)] | 2013 | ↑ | − |
| T-Lymphocyte apoptosis | Lim, S.C., et al. [[61](#_ENREF_61)] | 2011 | ↑ | + |
| Toll-like receptor 4 | Chen, H., et al. [[46](#_ENREF_46)] | 2012 | ↓ | − |
| TSH | Shoukry, A., et al. [[44](#_ENREF_44)] | 2013 | ↓ | + |

Abbreviations: ↑ = biomarker increased during AECOPD, ↓ = biomarker decreased during AECOPD, ↔ = Biomarker showed no change during AECOPD, + = Statistically significant (P-value < 0.05), − = Not statistically significant (P-value > 0.20), ± = Borderline statistically significant (P-value = 0.05-0.20), AECOPD = acute exacerbation of chronic obstructive pulmonary disease, BPI = bactericidal permeability increasing protein, CCL = chemokine C-C motif ligand, CD = cluster of differentiation, EPO = erythropoietin, FSH = follicle stimulating hormone, HMGB = high mobility group box, Ig = immunoglobulin, IL = interleukin, LH = luteinizing hormone, LT = leukotriene, MFAP = microfibrillar associated protein, MMP = matrix metallopeptidase, MR-proANP = mid-regional prohormone of atrial natriuretic peptide, NO = neutrophil elastase, nitric oxide, RBP = retinol-binding proteins, sICAM = soluble intercellular adhesion molecule, sTNFR = soluble tumor necrosis factor receptor, suPAR = soluble urokinase-type plasminogen activator receptor, TEAC = Trolox equivalent antioxidant capacity, T3 = triiodothyronine, T4 = thyroxine, TSH = thyroid stimulating hormone.
