# Supplementary material for: A Systematic Review of Diagnostic Biomarkers of COPD Exacerbation
Source: PLoS One. 2016 Jul 19;11(7):e0158843. doi: 10.1371/journal.pone.0158843 (PMC4951145; doi:10.1371/journal.pone.0158843)
Supplement: S7 Table — (DOCX) [file pone.0158843.s008.docx]

S7 Table. Modified REMARK (mREMARK) scores breakdown for the 12 studies listed in Table 2

| **Reference** | **Total Score** | **Intro** | **Materials and Methods** | | | | | | | | | **Results** | | | | | | | | **Discussion** | |
| --- | --- | --- | --- | --- | --- | --- | --- | --- | --- | --- | --- | --- | --- | --- | --- | --- | --- | --- | --- | --- | --- |
|  |  | 1 | 2 | 3 | 4 | 5 | 6 | 7 | 8 | 9 | 10 | 11 | 12 | 13 | 14 | 15 | 16 | 17 | 18 | 19 | 20 |
| Bafadhel, M., et al. | 18 | X | X | X | X | X | X |  | X |  | X | X | X | X | X | X | X | X | X | X | X |
| Lacoma, A., et al. | 16 | X | X | X | X | X | X |  | X |  | X |  | X | X | X | X | X | X |  | X | X |
| Stolz, D., et al. | 15 | X | X |  |  | X | X | X | X |  | X |  | X | X | X | X | X | X |  | X | X |
| Jin, Q., et al. | 15 | X | X | X | X | X |  | X | X |  | X |  | X | X | X | X |  | X |  | X | X |
| Hurst, J.R., et al. | 14 | X | X |  | X | X |  | X | X |  | X | X |  | X | X |  | X | X |  | X | X |
| Gumus, A., et al. | 13 | X | X |  | X | X | X |  | X |  | X |  |  | X | X | X |  | X |  | X | X |
| Shakoori, T.A., et al. | 13 | X | X |  | X | X | X |  | X |  | X | X |  | X |  | X |  | X |  | X | X |
| Falsey, A.R., et al. | 12 | X | X |  | X | X | X |  |  |  |  | X | X | X |  | X |  | X |  | X | X |
| Quint, J.K., et al. | 11 | X | X |  | X |  | X |  |  |  | X | X | X | X |  |  |  | X |  | X | X |
| Pazarli, A.C., et al. | 10 | X | X | X |  | X |  |  |  |  | X | X |  | X |  |  |  | X |  | X | X |
| Phua, J., et al. | 10 | X | X |  |  | X | X |  |  |  | X | X |  | X |  |  |  | X |  | X | X |
| Adnan, A.M., et al. | 6 | X |  |  | X | X |  |  |  |  |  | X |  |  |  |  |  |  |  | X | X |

This table summarized the mREMARK scores in studies from Table 2. A score is given to a particular section when the study met the recommendations
